# The coming of age of digital technologies in global health within the Indian context: a review

**DOI:** 10.1186/s42506-024-00169-5

**Published:** 2024-09-09

**Authors:** Vivek N. Dileep

**Affiliations:** Department of Political Science, University of Hyderabad, Gachibowli, Hyderabad, Telangana 500046 India

**Keywords:** Digital health, Health policy, India, Global Health Initiatives

## Abstract

Digital approaches have been recognized as an essential instrument for improving health systems to fulfill the Sustainable Development Goals (SDGs) and the targets for universal health care. This review article discusses policy and regulatory developments in the arena of digital health, at the global level, with a particular focus on India. It also points out that there is a need for convergence among industry, policymakers, and civil society in addressing issues of privacy and accessibility to all individuals who require affordable and quality healthcare. For the best use of digital services, inter-sectoral collaboration is necessary to integrate organizational, human, financial, and technological resources.

## Introduction

In the health care sector and everyday life worldwide, technology and data are essential. Data and technology are crucial as health systems deal with escalating expectations to provide novel, better, and seamless treatments that are affordable for everyone. Digital technology can help to enhance public health, strengthen health care finance and reach disadvantaged communities. The future of health and health care is anticipated to be technology-embedded and data-linked due to the possibilities and risks of advancements like artificial intelligence [1]. 

## Global Initiative on Digital Health

On August 19, 2023, at the Health Minister’s Meeting of The Group of Twenty (G20) Summit, which was hosted by the Government of India, the World Health Organization (WHO) and the G20 India presidency unveiled the Global Initiative on Digital Health (GIDH). The new GIDH project, which is called “guide” for short, will function as a network and platform under the aegis of the WHO to assist in the execution of the *Global Strategy on Digital Health 2020–2025*. The WHO will act as the secretariat for the strategy’s implementation, bringing together international standards, best practices, and resources to hasten the transition of the digital health system. To achieve the Sustainable Development Goals (SDGs) and the targets for universal health coverage, digital methods have been acknowledged as a crucial tool for enhancing health systems [2].

In recent years, both the WHO and the European Union (EU) have prioritized initiatives aimed at harnessing the potential of digital health applications to improve healthcare delivery and outcomes worldwide. The WHO launched several important initiatives in this regard, including the Digital Health Atlas, which serves as a repository of digital health interventions across the globe, facilitating knowledge sharing and collaboration among stakeholders [3]. An exemplary initiative by the WHO was the development and implementation of the WHO COVID-19 Mobile Application, which provided real-time updates, guidelines, and self-assessment tools to help individuals and healthcare professionals navigate the challenges posed by the pandemic [4].

Similarly, the European Union (EU) has made significant strides in promoting digital health initiatives through various programs and policies. One notable initiative is the Digital Health and Care Action Plan, which outlines the EU’s strategy for harnessing digital technologies to empower citizens, improve health outcomes and drive innovation in healthcare [5]. Under this plan, the EU has launched projects such as the European Health Data Space, which aims to facilitate the secure exchange and interoperability of health data across borders to support research and innovation. Additionally, the EU-funded Digital Europe Program allocates resources to support digital health projects, including the development of digital health infrastructures and solutions [6]. An illustrative example is the European Commission's support for the development and deployment of COVID-19 contact tracing apps including the European Federation Gateway Service under the aegis of the European Centre for Disease Prevention and Control (ECDC), which played a crucial role in the region's response to the pandemic while safeguarding individual privacy and data protection [7].

## Digital Technology in Medicine

The use of digital technology has progressively and creatively entered the field of medicine. In both developed and developing nations, there are issues with healthcare delivery related to the high burden of infections and non-communicable illnesses, the lack of human resources, the unequal distribution of healthcare, the lack of individualized care, and the lack of adequate emergency preparedness. Technology advancements have enhanced access to medical information, patient outreach services, and communication. Patients increasingly participate more actively in making healthcare decisions, emerging as customers and doctors as service providers. Digitalization in health care has been made possible by technology research and development that was sparked by patient-centered healthcare. The SDGs call for ensuring that everyone has access to affordable, high-quality healthcare. This can be done by using technology in a way that is both efficient and affordable [8]. The digital-in-health approach leverages digital technologies to enhance healthcare delivery, improve patient outcomes, and streamline health system operations, integrating innovations like telemedicine, health apps, and data analytics into the core of healthcare practices [9].

## Aspects of Digital Health

Seth Frank first proposed the idea of “digital health” in 2000 [10]. According to Frank, the World Wide Web and other interactive media, along with the related portals, browsers, and specialized web-based applications used to access them, will have a greater, more positive impact on medical care than any previous information technology or communications tool. Digital health encompasses various aspects, such as telemedicine, medical informatics, and mHealth (mobile health). Beyond electronic data capture, which includes tools for remote data capture, storage and data exchange like wearable technology and the Internet of Things (IoT), its functions have expanded significantly. Telemedicine now offers affordable and convenient virtual access to doctors, tele-radiology improves image interpretation and real-time data reporting, as well as mobile-based applications for medications and healthcare, are increasingly utilized [11].

## Recommendations of Riyadh Declaration on Digital Health

Public health responses to the recent pandemic outbreaks were impeded by broad, ongoing underinvestment in digital health, as highlighted by COVID-19. Recognizing this issue, the Riyadh Declaration on Digital Health [12], developed by an international interdisciplinary team of medical, academic, and industry experts at the Riyadh Global Digital Health Summit in August 2020, provided a series of digital health recommendations for the global health community to address the challenges of current and upcoming pandemics. However, guidance on how to put these suggestions into practice is still needed.

## WHO Recommendations and Resolutions in Digital Health

Early in the development of digital health, there was little financial backing and enthusiasm for rigorously evaluating the effects of these interventions [13]. The initial possible sense of digital exceptionalism has since been significantly diminished, and a solid body of research addressing various aspects of digital health has been disseminated. The WHO mHealth Technical Evidence Review Group developed new recommendations [14] and a toolkit [15] in 2016 to enhance and standardize the research and reporting of mHealth in the literature. This effort was done to support the development and promotion of high-quality digital health research. The new recommendations include a checklist that is designed to establish a basic set of information required to delineate the nature of the mHealth intervention (content), its implementation location (context), and the methodology of its implementation (technical features), thereby facilitating the replication of the intervention. The toolkit helps researchers and policymakers navigate the process of developing value “claims,” selecting indicators and evaluation designs for their digital health interventions, assessing the quality and availability of intervention data, and providing guidelines for reporting findings.

A resolution on digital health that states “governments must acknowledge the relevance of digital technologies for aiding the improvement of health systems and achieving universal health coverage” was unanimously adopted by WHO member states in May 2018 at the 71st World Health Assembly. According to the resolution, there is a need to “ensure that digital health solutions complement and enhance existing health service delivery models” [16], strengthen patient-centered health services that are already integrated, contribute to improving population health and gender and health equity, and address the scarcity of research and evidence on the effects of digital health on public and clinical health. To ensure that the full promise of digital health is realized, the resolution identifies coordinated, methodical, and evidence-based measures that the WHO, with member states and the larger ecosystem of partners, need to prioritize. The historic May 2018 WHO meeting on the development of digital health guidelines and recommendations on digital interventions for strengthening health systems marks a turning point in the digital health ecosystem. This meeting addressed a crucial convergence point in the discourse on health systems strengthening and digital health innovation by consolidating the evidence base in the form of global recommendations [17].

## Global Strategy on Digital Health

Further, the *Global Strategy on Digital Health 2020–2025* was unveiled by the WHO in 2021 with the vision to “to improve health for everyone, everywhere by accelerating the development and adoption of appropriate, accessible, affordable, scalable and sustainable person-centric digital health solutions to prevent, detect and respond to epidemics and pandemics, developing infrastructure and applications that enable countries to use health data to promote health and well-being, and to achieve the health-related Sustainable Development Goals and the triple billion targets of WHO’s Thirteenth General Program of Work, 2019–2023” [18]. To make it more inclusive, the document defines digital health as “the field of knowledge and practice associated with any aspect of adopting digital technologies to improve health.” The strategy aims to assist nations in maximizing the use of digital healthcare technology in a sustainable, equitable, accessible, and scalable manner so that it improves patient health management, fosters better communication with healthcare providers, and aids nations in tracking the effects of health policies for future advancement.

The Global Strategy also places a strong emphasis on the requirement for inter-sectoral collaboration to integrate organizational, human, financial, and technological resources for the greatest possible use of digital services. Future expectations for digital health include better patient care through resource management that is more effective. Countries can incorporate information and technology in their healthcare systems with the aid of the National eHealth plan toolkit [19] developed by the WHO.

## India’s Digital Health Landscape

Several digital health initiatives have already been adopted by the Government of India, including the mother and child tracking system, hospital information system, drugs and vaccines distribution system, and ASHA-Soft [20] (a website developed by the Government of Rajasthan to gather beneficiary data for each Accredited Social Health Activist, or ASHA,^1^ and managing their incentives accordingly). Further, the Government of India has established the National Health Portal [21] to provide access to medical information with integrated services like online registration, information about the closest medical facility, information, education and communication materials, personalized health records, mHealth, telemedicine, eRaktKosh (a project to network blood banks nationwide, digitize their processes, and streamline their operations) and details about AYUSH^2^/naturopathy and other curative services.

Under the goals of the National Health Policy of 2017, the Ministry of Health and Family Welfare published the National Digital Health Blueprint in April 2019 [22]. The architectural document is an action plan to support the district-level digitalization of health data, the upkeep of major illness registries, and the connection of primary healthcare services and referral services. Other important elements in the document include the development of a distinctive digital health ID, medicine supply chain management, payment gateways, and the establishment of norms and regulations within the operating framework for patient safety, service quality, privacy, and data security.

Crucially, the Government of India launched the Ayushman Bharat Digital Mission (ABDM) in September 2021 [23]. The ABDM aims to develop a seamless online platform by offering a variety of data, information, and infrastructure services while appropriately utilizing open, interoperable, standards-based digital systems to ensure the security, confidentiality and privacy of personal health information. With their permission, the residents’ longitudinal health records will be accessible and exchangeable through the Mission. The ABDM’s main elements include a health ID for each citizen that will also serve as their health account, to which individual health records can be linked and viewed with the aid of a mobile application, as well as a Healthcare Professionals Registry (HPR) and Healthcare Facilities Registries (HFR) that will serve as a repository of all healthcare providers from both allopathic and traditional medical systems. This will make it easier for hospitals, doctors, and other healthcare service personnel to provide services.

At the state level too, many Indian state governments have made considerable progress towards launching digital health projects, including Kerala, Tamil Nadu, and Rajasthan. Tamil Nadu was one of the first states to launch a digital health initiative in 2008 [24]. With the help of the World Bank, a thorough state-wide health management information system (HMIS) was created to expedite clinical, logistical, and administrative procedures thereby increasing the operational effectiveness of the state’s public health system. Similarly, Kerala led the way in the healthcare sector’s digitization by introducing the e-Health Kerala program in 2017 [25]. Through this system, each patient receives a special ID facilitating the compilation and connection of the patient's whole medical history.

Additionally, large and small firms in the health technology industry have benefited from the wave of digitization in recent years as the private sector has advanced significantly. The health market has grown largely due to the quick uptake of smartphones and the internet, supportive government regulations, and other factors. According to estimates, India’s digital healthcare market was worth USD 6.3 billion in 2021. By 2027, it is estimated to grow by 28.50% from 2022 to 2027, reaching USD 30 billion [26]. However, the digital health sector has been hampered by severe fragmentation, a lack of communication, inadequate data portability, and uncoordinated efforts to create a digital health architecture for India.

## India’s digital health response: navigating the COVID-19 pandemic

India has witnessed significant advancements in digital health applications in recent years, particularly in the context of healthcare delivery. The COVID-19 pandemic catalyzed the adoption and utilization of digital health technologies across the country, enabling a nationwide response of unprecedented scale. One notable development was the implementation of nationwide dashboards such as the *COWIN* to track key indicators such as morbidity, mortality, and immunization coverage in real-time [27]. These dashboards, developed by government agencies and private organizations, provided policymakers, healthcare professionals, and the public with timely and accurate data to inform decision-making, allocate resources effectively, and monitor the spread of the virus. Additionally, digital health platforms such as *Aarogya Setu* [28], launched by the Government of India, played a crucial role in facilitating contact tracing, risk assessment, and self-reporting of COVID-19 symptoms, empowering individuals to protect themselves and others while contributing to national efforts to contain the spread of the virus.

Furthermore, the pandemic spurred innovation and collaboration in the digital health ecosystem, leading to the development of telemedicine platforms, remote monitoring solutions, and virtual care services to ensure continuity of healthcare delivery amid lockdowns and social distancing measures. Teleconsultation initiatives such as *Sehat* and *e-Sanjeevani* surged in popularity, allowing patients to access healthcare services remotely and reducing the burden on overstretched healthcare facilities [29]. Public–private partnerships and initiatives by start-ups and technology companies further accelerated the adoption of digital health solutions, bridging gaps in healthcare access and delivery, particularly in underserved regions [30]. The unprecedented scale and impact of India's digital health response to the COVID-19 pandemic underscore the transformative potential of technology in healthcare delivery and public health management, laying the foundation for a more resilient and inclusive healthcare system in the country.

## Digital health landscapes: comparing India with LMICs

India’s digital health landscape offers valuable insights compared to other low- and middle-income (LMIC) countries such as South Africa, Brazil, and Bangladesh. While each country faces unique challenges and opportunities, India stands out for its large population and diverse healthcare needs, driving the scale and complexity of digital health initiatives. In contrast, countries like South Africa, Brazil, and Bangladesh have smaller populations but grapple with similar issues of healthcare accessibility, infrastructure, and resource constraints [31]. Despite these differences, all four countries have made strides in leveraging digital technologies to improve healthcare delivery, with varying degrees of success. South Africa, for example, has implemented telemedicine and mHealth solutions to address gaps in healthcare access, particularly in rural and underserved areas [32]. Brazil has pioneered digital health platforms for electronic health records and teleconsultations, enhancing care coordination and patient engagement [33]. Bangladesh, on the other hand, has focused on mobile health interventions to deliver healthcare services and health information to remote populations, overcoming geographical barriers and improving health outcomes [34].

However, challenges persist in the adoption and implementation of digital health applications across these countries, including issues related to infrastructure, internet connectivity, digital literacy, and data privacy [35]. While India has made significant progress in digital health infrastructure and innovation, disparities in access to technology and healthcare persist, particularly among rural and marginalized communities. Similarly, South Africa, Brazil, and Bangladesh face barriers to widespread digital health adoption, including inequitable access to healthcare services, regulatory constraints, and funding limitations [36]. Nevertheless, by sharing best practices, lessons learned and innovative solutions, these countries can collaborate to overcome common challenges and harness the transformative potential of digital health to advance universal health coverage and improve health outcomes for all.

## Charting the course: challenges and opportunities in digital health

Despite its promise, digital health poses several challenges. Patients who obtain medical information from unreliable online sources and dubious websites may misinterpret it. Data security and privacy issues arise because medical devices can be susceptible to hacking and data loss [37]. Delivering healthcare solely through a digital platform is impersonal and undermines the doctor-patient relationship's confidence. Ethics in practice are also threatened by a lack of human resource training in using application and interpreting technology.

Digital health services must be implemented with solid governance to protect data privacy and effective resource management that adheres to the highest societal and ethical standards. The European Public Health Alliance emphasized the importance of involving primary and secondary healthcare providers, including pharmacies, and suggested that digital health be positioned as an addition to conventional healthcare rather than a replacement for it [38]. Similar obstacles will need to be identified and addressed as the National Digital Health Mission in India is implemented.

Health information, education, communication, monitoring, diagnostics and data processing are all set to be revolutionized by digital health. For nations to create, implement and collaborate on these services in the best interest of individuals, the WHO Global Strategy offers a framework. The National Digital Health Mission can further aid in developing and providing the best personalized digital health ecosystem for India.

When it comes to the ongoing transformation of health systems and how healthcare is provided to everybody, digital solutions, and data are strong accelerators. However, the present strategies are fragmented, sporadic, and opportunistic [39].

## Conclusion

Digital initiatives have received official recognition as a vital approach for achieving the SDGs and the targets for universal health care. To gain more value out of technology and data and guarantee that health systems deliver treatment that is accessible, egalitarian, and cost-effective, new thinking—a digital-in-health approach—is required. By integrating technology and data across numerous facets of health systems, including financing, service delivery, nutrition, research, pandemic preparedness, and medical education, digital-in-health goes beyond the common definition of digital health. Health services can be made more accessible, effective economical, and relevant to the people by integrating digital components into every aspect of health systems and their management.

Further, governments have a crucial role in fostering trust in and capacity for the use of digital technology in health systems, promoting equity and bridging rather than widening the digital divide. Governments, the private sector, and civil society need to lead this shift, guiding healthcare towards a future powered by data and the internet. There will be difficulties in navigating these innovative waters. However, with the appropriate approach—one that includes effective national leadership, sound health data governance, private sector collaboration, evidence-based decision-making, a focus on interoperability and broader digital transformation across government systems and in society, as well as a discerning focus on sustainability—the promised value of digital technologies can be realized for everyone.

## Data Availability

Not applicable.

## Footnotes

1 Accredited Social Health Activist, or ASHA, worker is female healthcare activist trained to facilitate the work of the nationwide public health system within their community. ASHA workers are a core part of the National Rural Health Mission launched by the Government of India.

2 AYUSH is an acronym for Ayurveda, Yoga and Naturopathy, Unani, Siddha and Homeopathy and are the six Indian systems of medicine prevalent and practiced in India and some of the neighbouring South Asian countries.
